# PKM2 Is the Target of a Multi-Herb-Combined Decoction During the Inhibition of Gastric Cancer Progression

**DOI:** 10.3389/fonc.2021.767116

**Published:** 2021-12-02

**Authors:** Qingmin Sun, Mengyun Yuan, Hongxing Wang, Xingxing Zhang, Ruijuan Zhang, Haidan Wang, Xu Chen, Min Zhu, Shenlin Liu, Jian Wu

**Affiliations:** ^1^ Jiangsu Province Hospital of Chinese Medicine, Affiliated Hospital of Nanjing University of Chinese Medicine, Nanjing, China; ^2^ No. 1 Clinical Medical College, Nanjing University of Chinese Medicine, Nanjing, China

**Keywords:** traditional Chinese medicine, PKM2, gastric cancer, aerobic glycolysis, cancer metabolism

## Abstract

Gastric cancer is the third leading cause of cancer death worldwide. Traditional Chinese medicine (TCM) is increasingly extensively applied as a complementary therapy for gastric cancer (GC) in China, which shows unique advantages in preventing gastric cancer metastasis. Previous study indicates modified Jian-pi-yang-zheng (mJPYZ) decoction inhibit the progression of gastric cancer by regulating tumor-associated macrophages (TAM). However, it is unclear whether mJPYZ can affect metabolic reprogramming of gastric cancer cells. Here, we showed that mJPYZ effectively attenuated GC cells proliferation, migration and invasion. Meantime, mJPYZ reduced the aerobic glycolysis level of GC cells *in vivo* and *in vitro* by regulating the expression and nuclear translocation of PKM2. Overexpression of PKM2 that could reverse the inhibitory effect of mJPYZ, migration and epithelial to mesenchymal transition (EMT). Our results showed PKM2/HIF-1α signaling was the key metabolic regulator of mJPYZ in GC cells. In summary, our present study suggested that abnormal PKM2 is required for maintaining the malignant phenotype of GC cells. The TCM decoction mJPYZ inhibited GC cells growth and EMT by reducing of glycolysis in PKM2 dependent manner. This evidence expanded our understanding of the anti-tumor mechanism of mJPYZ and further indicated mJPYZ a potential anti-tumor agent for GC patients.

**Chemical Compounds Studied in this Article:**

Rutin (PubChem CID: 5280805); Lobetyolin (PubChem CID: 53486204); Calycosin-7-glucoside (PubChem CID: 71571502); Formononetin (PubChem CID: 5280378); Calycosin (PubChem CID: 5280448); Ononin (PubChem CID: 442813); P-Coumaric Acid (PubChem CID: 637542).

## Introduction

According to the latest global cancer statistics, gastric cancer is still the fifth most commonly diagnosed cancer and the fourth leading cause of cancer mortality worldwide in 2020 (1). There is an urgent need to develop strategy to prevent the recurrence and metastasis of gastric cancer patients. Traditional Chinese medicine (TCM) has been well accepted and is increasingly extensively applied as a complementary and alternative therapy for gastric cancer (GC) in China, which show unique advantages in preventing and treating gastric cancer metastasis (2). Our previous study showed that Jianpi Yangzheng Xiaozheng (JPYZXZ) decoction could significantly improve life quality and prolong the survival of patients with advanced stage gastric cancer (3). Our recent study further indicated that the components of JPYZXZ also inhibited the progress of gastric cancer *in vivo* and *in vitro* (4). Moreover, mechanism research showed modified Jianpi Yangzheng decoction (mJPYZ) alleviates gastric cancer progression *via* macrophages immune checkpoint PI3Kγ (5). However, it is unclear whether mJPYZ can affect metabolic reprogramming of gastric cancer cells.

Cell metabolic reprogramming exist in various cancer cells and are implicated as salient hallmarks of cancer (6). “Aerobic glycolysis” or “Warburg effect”, as a classic metabolic reprogramming pathway of tumor cells, refers to the fact that cancer cells give priority to glycolysis instead of oxidative phosphorylation (OXPHOS) to produce energy under normoxic conditions (7). Numerous studies have shown that aerobic glycolysis is closely related to occurrence, metastasis, and poor prognosis of gastric cancer (8, 9). Meanwhile, in the tumor microenvironment, lactate, a by-product of glycolysis, can also facilitate tumor immunosuppression (10). Mechanistically, a variety of oncogenes and their targeted metabolic enzymes, including phosphofructokinase (PFK), hexokinase (HK), lactate dehydrogenase (LDHA), and pyruvate kinase1/2 (PKM1/2), can promote glycolysis, resulting in cancion genesis, progression, and metastasis. Therefore, target glycolysis has become a novel therapeutic strategy for the treatment of gastric cancer.

PFK, HK, PK are three important rate-limiting enzymes in aerobic glycolysis. Among them, PK is considered to be the central kinase that reprograms cell metabolism, which catalyzes the conversion of phosphoenolpyruvate to pyruvate (11).

There are four isoforms of the PK protein family, PKM1, PKM2, PKL, and PKR. PKM2 has been revealed to be dramatically increased in gastric cancer cells. When PKM2 is deleted in GC cells, the PI3K/AKT/mTOR pathway and autophagy are inhibited, resulting in a decrease in the proliferation and invasion phenotype of GC cells (8, 12). Specially, studies have demonstrated that PKM2 promote transcription of HIF-1α to enhance glycolysis by translocate to the nucleus (13).

Recent studies indicate that TCM have a regulatory effect on glucose metabolism, and metabolism-related factors and enzymes may be their targets (14). mJPYZ decoction contains Astragalus mongholicus (Huangqi); Codonopsis pilosula (Dangshen); Sparganium stoloniferum (Sanleng); Curcuma phaeocaulis (Erzhu), which is an optimized version compared to JPYZXZ and could inhibit gastric cancer growth and metastasis (4). In the current study, we hypothesized that mJPYZ could inhibit PKM2 and remodel aerobic glycolysis in GC cells. We elucidated the potential mechanism of mJPYZ decoction in reducing the aerobic glycolysis level and alleviating the progression of gastric cancer.

## Materials and Methods

### Cell Culture

The MGC-803, SGC-7901 and BGC-823 human gastric cancer cell lines were obtained from the Cell Bank of Type Culture Collection of the Chinese Academy of Sciences (Shanghai, China), and were cultured in RPMI 1640 supplemented with 10% Newborn calf serum in a humidified incubator at 37°C with 5% CO_2_.

### Preparation of mJPYZ

All herbs were supplied by SANYUE Chinese Traditional Medicine Co. (Nantong, China) and were identified by a Chinese pharmacist. The herbs were soaked and boiled with double-distilled water for 30 min, then the extracted solutions were quantified to 1 g/mL. The extract was stored at −20°C after sterilization and filtering by a 0.22 μm filter.

### MTT Assay

GC cells (5 × 10^3^ cells/well) were seeded into 96-well plates separately for 24 h to allow adherence to the walls. To determine the decoction dose, the IC50 of the MGC-803, SGC-7901 and BGC-823 was calculated and the dose range of 2, 4, 8 mg/mL was selected. Then mJPYZ decoction was added at different concentrations (0, 1, 2, 4, 8, 16 mg/mL) for 24, 48, 72 h. MTT (120 μL, 5 mg/mL) (Sigma, USA) was added after the medium was removed, and the cells were incubated for 4 h in the incubator. The supernatant was removed, and 150 μL of dimethyl sulfoxide (DMSO) was added for 10 min. Absorbance at 490 nm was detected on an ELX800 Automatic microplate reader (Bio-Tek, USA) to calculate the absorption value.

### Colony Formation

GC cells were seeded at 2000 cells/well in 6-well plates. After a 24h cells adherence, mJPYZ decoction was added to treat the cells for another 10 days. At the end of treatment, the cells were fixed with 95% ethanol and stained with 0.1% Crystal Violet. Photos of each group were taken under a microscope, and the number of colonies formed was then counted.

### Wound-Healing Assay

GC cells (5 × 10^5^ cells/well) were seeded into 6-well plates, and a straight line was drawn on the bottom of the plates with a pipette tip when 80% of the cells were adherent to the walls. The plates were washed lightly with PBS 3 times. mJPYZ decoction with different concentrations were set into the wells, and pictures were taken again after incubation for 48 h, and the area between wound boundaries was recorded.

### Transwell Invasion Assay

Matrigel (100 μL, diluted 1:29 with PBS) (Corning, USA) was vertically added to the inside bottom of the 8-μm Transwell chambers (Merck & Millipore, Germany), and the chambers were dried 1 h before use. After a 24 h serum withdrawal, cells (2 × 10^5^ cells/mL) were added to the chamber, and mJPYZ decoction with different concentrations were set into the 24-well plates. After 48 h, the migrated cells were fixed with 95% ethanol and stained with crystal violet. Photos of each group were taken under a microscope, and sections were counted randomly.

### Flow Cytometry Analysis of Apoptosis and Cell Cycle

Cells were seeded in 6-well plates for 24 h, and treated with 0, 2, 4, and 8 mg/mL mJPYZ for 48 h. Cells were then harvested and stained with annexin V-FITC and PI, and detected by flow cytometry. For cell cycle, cells were then harvested and fixed with 75% ethanol, and was determined using propidium iodide (PI) staining and FACSAria III flow cytometer (BD Biosciences, USA) analysis according to the manufacturer’s protocol.

### Western Blotting Analysis

Total protein was extracted from cells using RIPA lysis buffer. The nuclear and cytoplasmic protein was extracted using a commercial kit (Yeasen Biotechnology, Shanghai, China). The protein concentration was determined with a BCA kit. Different groups of protein were separated using sodium dodecyl sulfate polyacrylamide gel electrophoresis (SDS-PAGE) and transferred to polyvinylidene difluoride (PVDF) membranes (Millipore, Billerica, MA, USA). After blocking with 5% nonfat milk for 1 h at room temperature, the membranes were incubated with 1:1000 primary antibodies E-cadherin (Cell Signaling Technology, #3195), N-cadherin (Proteintech, 22018-1-AP), Vimentin (Cell Signaling Technology, #5741), MMP-9 (Cell Signaling Technology, #13667), MMP-2 (Cell Signaling Technology, #40994), β-Actin (Cell Signaling Technology, #8457), HK2 (Proteintech, 22029-1-AP), PFKFB3 (Proteintech, 13763-1-AP), PDK1 (Cell Signaling Technology, #3062), PDK4 (Abcam, ab89295), PKM2 (Proteintech, 15922-1-AP), HIF-1α (Bioss, bs-0737R/Abcam, ab51608), Lamin B1 (Proteintech, 12987-1-AP) at 4°C overnight. Then the membranes were incubated with secondary antibodies at room temperature for 1 h. The membranes were finally scanned with a Bio-rad ChemiDoc XRS+ (Berkeley, CA, USA).

### Co-Immunoprecipitation (Co-IP) Assay

To analyze interactions of HIF-1α protein with related proteins, cells seeded in 10-cm-diameter culture dishes were lysed, followed by centrifugation at 15,000 ×g for 15 min. Supernatants were precipitated with Rabbit IgG or HIF-1α specific Polyclonal antibody (Abcam, Cambridge, UK) and incubated with gentle rocking overnight at 4°C. Protein A/G beads washed with cell lysate were added to supernatant fractions and incubated with gentle rocking for 12 h at 4°C. The beads were then washed four times with cold cell lysate and boiled with 1 × SDS loading buffer for 10 min, followed by SDS-PAGE. The same ways to analyze interactions of HIF-1α protein with related proteins.

### Biochemical Assays

The lactate concentration of the cell supernatant or the plasma were determined using a Lactate Testing Kit (Jian cheng Bioengineering Institute, Nanjing, China), according to the manufacture’s protocol. And the glucose concentration of the cell supernatant or the plasma were determined using a Glucose Assay Kit (Rongsheng Biotechnology, Shanghai, China), according to the manufacture’s protocol.

### Immunofluorescence Staining

Cells were seeded in glass coverslips for 24 h, and treated with 8mg/mL mJPYZ for 48 h. The treated cell slices were fixed with 4% paraformaldehyde, washed with PBS, and incubated with primary antibodies against PKM2 at 4°C overnight. The next day, after incubation with fluorescence secondary antibody and 4′,6-diamidine-2-phenylindole, the slices were mounted and observed with an FV1000 laser scanning confocal microscope (Leica, Wetzlar, Germany). All double immunofluorescence images were captured under the same conditions. The co-localization analysis was performed using Image J software (NIH Image, Bethesda, MD, USA).

### Plasmid Construction, Lentivirus Packaging, and Cell Transfection

The PKM2 overexpression lentivirus was synthesized by Shanghai Engineering Research Center of Cancer Drug Targets (Shanghai, China). Briefly, to create a recombinant plasmid overexpressing PKM2-OE, a full-length cDNA encoding the PKM2 sequence was amplified and cloned into the GV358 vector, which contained the component order Ubi-MCS-3FLAG-SV40-EGFR-IRES-puromycin. Then the recombinant plasmid was under identification and sequencing analysis. Finally, after plasmid extraction, got the highly-purified plasmid.

To establish a stable lentivirus transfection GC cell line, MGC-803, SGC-7901 and BGC-823 cells were seeded in 6-well plates, and when 60–70% confluent, they were transfected with PKM2-OE lentivirus in the presence of 5 μg/mL polybrene for 24 h. The positive cells were selected by puromycin and transfection efficiency was determined by inverted fluorescence microscope and western blotting.

### Establishment of Xenograft Tumor Model

Mice (615-strain) were purchased from the Tianjin Institute of Blood (6 weeks old, weighing 18−22 g). All mice were maintained under standard conditions of 50% relative humidity, 21 ± 2°C, and a 12 h light cycle. Each mouse was inoculated with 0.2 mL of cell suspension (5 ×10^7^/mL, MFC murine gastric cancer cells)in the right armpit after disinfection. The diameter of the induration reached 3−7 mm after 10 days, which shows the establishment of a successful model. Fifteen mice were divided randomly into 3 groups: control group (normal saline, NS 0.2 mL/10 g *via* gavage), 5-Fluorouracil (5-Fu) group (5-Fu 25 mg/kg *via* intraperitoneal injection) and mJPYZ group (mJPYZ 15g/kg, 0.2mL/10 g *via* gavage). All the mice were medicated for 14 days (5-Fu intraperitoneal injection every other day) continuously. The weight of mice was recorded every 4 days and sacrificed on day 21. Tumors were excised from the animals and weighed immediately.

Mice (BALB/c-nu) were also purchased from the Tianjin Institute of Blood (6 weeks old, weighing 18−22 g). After adaptive culturing, nude mice were randomly divided into negative control (NC) group, PKM2 overexpression (PKM2-OE group) group and PKM2 overexpression with mJPYZ mediated (PKM2-OE+mJPYZ) group. SGC-7901 cells with stable expression of PKM2 were taken, cellular concentration was adjusted to 1 × 10^7^ cells and they were inoculated beneath right axilla skin of each nude mouse with the inoculation density being 0.2ml. The growth status of subcutaneous xenograft in nude mice was closely observed.

The Experimental Animal Ethics Committee of Nanjing Medical University approved all animal experiments.

### Extracellular Acidification Rate (ECAR) and Glycolysis Proton Efflux Rate (glycoPER)

Assays were performed using a Seahorse XFe24 analyzer (Seahorse Bioscience, Agilent Technologies) according to the Manufacturer’s instructions. The ECAR was measured using a Seahorse XF Glycolytic Rate Assay Kit (Seahorse Bioscience, Agilent, 103344-100). The glycolytic capacities of cells were analyzed with the Seahorse XF Glycolysis Rate/Stress Test Report Generator package. The %PER from glycolysis was calculated by subtracting the acidification from CO_2_ produced by the mitochondria, also called glycoPER.

### Statistical Analysis

Each experiment was repeated at least three times. All quantitative data are expressed as the mean ± standard deviation (SD) and analyzed using GraphPad Prism 7.0 software (GraphPad Software, San Diego, CA, USA). The comparisons between two groups were analyzed by Student’s t-tests (unpaired, two-tailed) or the one-way analysis of variance (ANOVA) (followed by Tukey’s *post-hoc* tests). Statistical significance was defined as P < 0.05.

## Results

### mJPYZ Inhibited Gastric Cancer Cells Proliferation, Migration and Invasion

**Figure 1:**
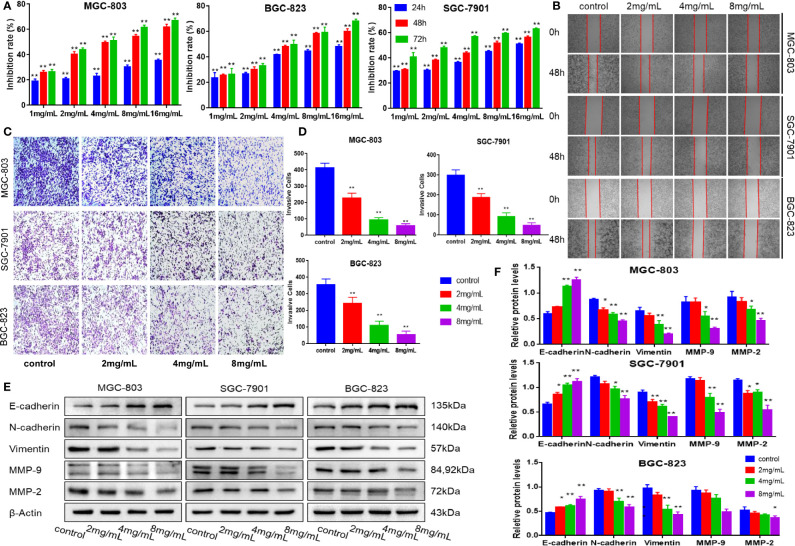
mJPYZ inhibited the proliferation, migration and invasion of gastric cancer cells *in vitro*. **(A)** Viability of cancer cells interfered by mJPYZ was determined using the MTT assay. mJPYZ inhibited the GC cells proliferation in a dose- and time-dependent manner. **(B)** Migration of gastric cancer cells were detected by wound healing assay. The cell migration ability was decreased after mJPYZ treatment, and was positively correlated with the concentration of mJPYZ. **(C, D)** Invasion of gastric cancer cells were detected by Transwell invasion assay. The cell invasion ability was decreased after mJPYZ treatment, and was positively correlated with the concentration of mJPYZ. **(E, F)** Western blotting analysis of fundamental proteins related to EMT. mJPYZ treatment increased E-cadherin expression, while it decreased the expression of N-cadherin, Vimentin, MMP-9 and MMP-2 in a dose-dependent manner. Data are represented as mean ± SD, n=3 independent experiments. *P < 0.05, **P < 0.01 versus control group by one-way analysis of variance (ANOVA) followed by Tukey’s *post-hoc* tests.

MTT assay was used to determine the toxicity of mJPYZ on GC cell lines (MGC-803, SGC-7901, BGC-823), As shown in **Figure 1A**, mJPYZ inhibited the GC cells proliferation in a dose- and time-dependent manner. Meantime, the results of Scratch-healing showed that the wound healing speed of GC cells was significantly reduced when mJPYZ treatment, indicating that the cell migration ability was decreased, and it was positively correlated with the concentration of mJPYZ (**Figure 1B**). Transwell assay also confirmed that mJPYZ could inhibit the migration and invasion of gastric cancer (**Figures 1C, D**). Moreover, Western blot analyses of E-cadherin, N-cadherin, Vimentin, MMP-2 and MMP-9, which were all metastasis related proteins, suggested mJPYZ significantly inhibited EMT and invasion of GC cells (**Figures 1E, F**). Taken together, these results showed that mJPYZ significantly inhibited GC cells proliferation, migration and invasion.

### mJPYZ Decreased Tumor Load and Metastasis in a Xenograft Tumor Model

**Figure 2:**
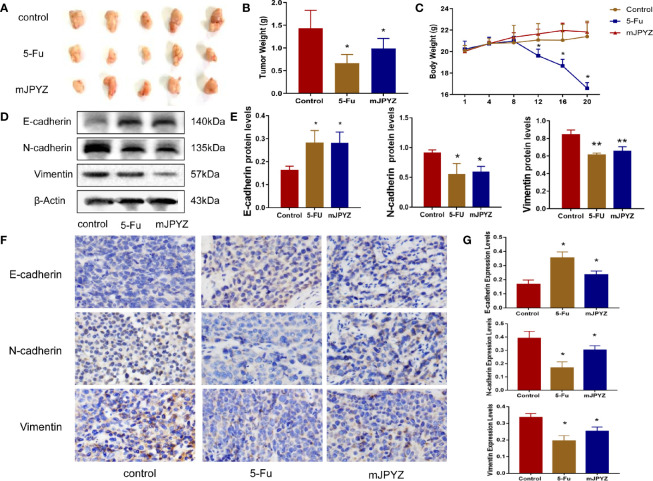
mJPYZ decreased tumor growth and metastasis *in vivo*. **(A)** The gross manifestation and volumes of tumors. **(B)** The tumor weight. **(C)** The weight of the mice which recorded every 4 days. Effect of mJPYZ on the growth of SGC-7901 xenograft tumors (n = 5). **(D, E)** Western blotting analysis xenograft tumor tissues of essential proteins related to EMT. **(F, G)** Immunohistochemistry showed the rates of E-cadherin, N-cadherin and Vimentin expression. Compared to control, mJPYZ treatment increased the levels of E-cadherin, while decreased the levels of N-cadherin and Vimentin. Data are represented as mean ± SD, n=5 mice or n=3 independent experiments. *P < 0.05, **P < 0.01 versus control group by one-way analysis of variance (ANOVA) followed by Tukey’s *post-hoc* tests.

Next, we established a xenograft tumor model using SGC-7901 GC cells to verify the effect of mJPYZ *in vivo*. As shown in **Figures 2A, B**, the treatment groups exhibited decreased tumor volume compared to the NC group after 15g/kg mJPYZ per day administration. The weights of the mice treated with 5-Fu was significantly reduced, while mJPYZ groups had no statistical difference (**Figure 2C**). Similar to the results of *in vitro*, we also found mJPYZ increased the protein expression levels of E-cadherin and decreased the levels of N-cadherin, Vimentin, which indicated that mJPYZ inhibited gastric cancer growth and EMT in the xenograft tumor (**Figures 2D–G**). These data suggested that mJPYZ controlled GC cells growth and metastasis by modulating EMT *in vivo*.

### mJPYZ Reduced the Aerobic Glycolysis Level in GC Cells

**Figure 3:**
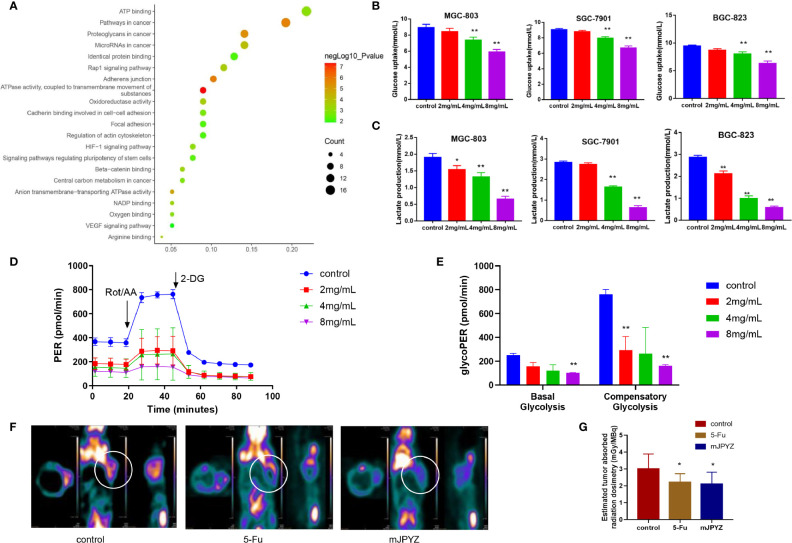
The effects of mJPYZ on EMT of gastric cancer cells depended on aerobic glycolysis. **(A)** Network pharmacology and Gene Oncology (GO) analysis investigated mJPYZ corresponding pathways participate in progress of gastric cancer and found related to aerobic glycolysis. **(B, C)** The effect of mJPYZ on the glucose uptake and lactate production levels in gastric cancer cells. **(D, E)** Extracellular acidification rates (ECAR) and Proton Efflux Rate (PER) were measured in SGC-7901 cells by Seahorse XFe assays. Quantification of the PER from glycolysis of mJPYZ with control group. mJPYZ administration decreased ECARs, PER, glucose consumption and lactate production in GC cells compared with control group. **(F, G)** MicroPET-CT on tumor-bearing nude mice. After mJPYZ treatment, 18F-fluorodeoxyglucose (^18^FDG) accumulation was reduced in tumor. Data are represented as mean ± SD, n=3 independent experiments. *P < 0.05, **P < 0.01 versus control group by one-way analysis of variance (ANOVA) followed by Tukey’s *post-hoc* tests.

To determine the underlying mechanism of mJPYZ on GC cells, we used network pharmacology and Gene Oncology (GO) analysis to investigate mJPYZ corresponding pathways participate in progress of gastric cancer. The results suggested that underlying modulate mechanism of mJPYZ on GC cells including cellular energy metabolism, HIF-1αsignaling etc. (**Figure 3A**). Previous studies have shown that robust aerobic glycolysis is associated with tumor invasion and metastasis. Therefore, we explored whether the anti-tumor activity of mJPYZ is related to aerobic glycolysis. As shown in **Figures 3B, C**, mJPYZ treatment for 48 h significantly inhibited glucose uptake and supernatant lactate levels in a dose-dependent manner. Moreover, mJPYZ administration decreased extracellular acidification rates (ECARs), glucose consumption and lactate production in GC cells compared with control group. Consistently, both basal glycolysis and compensatory glycolysis were abrogated by mJPYZ (**Figures 3D, E**). Additionally, microPET-CT images also confirmed the reduced ^18^F-fluorodeoxyglucose (^18^FDG) accumulation on tumor-bearing nude mice of mJPYZ treatment (**Figures 3F, G**). Therefore, target glucose metabolism may be a potential mechanism of mJPYZ in treatment of gastric cancer.

### mJPYZ Inhibited the Expression and Nuclear Translocation of PKM2 in GC Cells

**Figure 4:**
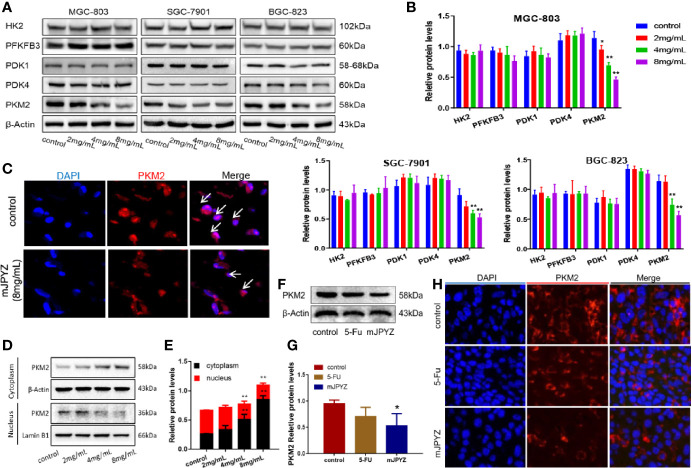
mJPYZ effected the expression and location of PKM2 in gastric cancer. **(A, B)** Western blotting analysis of three rate-limiting enzymes and ATP related enzymes. mJPYZ treatment decreased the expression of PKM2 in a dose-dependent manner, while the others expression unchanged. **(C)** Immunofluorescence showed PKM2 expression was reduced after mJPYZ treatment *in vitro*. **(D, E)** mJPYZ suppressed PKM2 translocating into the nucleus, meanwhile increased in the cytoplasm. **(F, G)** Western blotting analysis of PKM2 expression *in vivo*. mJPYZ treatment decreased the expression of PKM2. **(H)** Immunofluorescence showed PKM2 expression was reduced after mJPYZ *in vivo*. Data are represented as mean ± SD, n=3 independent experiments. *P < 0.05, **P < 0.01 versus control group by the one-way analysis of variance (ANOVA) followed by Tukey’s *post-hoc* tests.

Given the rate-limiting enzymes role of HK2, PFKFB3 and PKM2 in aerobic glycolysis, we investigated the effect of mJPYZ on three key enzymes. Moreover, we observed the expression of pyruvate dehydrogenase kinases (PDKs), which dominate the synthesis of biological macromolecules in the glycolysis of tumor cells. Western blot results showed that mJPYZ inhibited the expression of PKM2, which catalyses the last step of glycolysis, but had no obvious effect on the levels of HK2, PFKFB3, PDK1 and PDK4 (**Figures 4A, B**). Similarly, immunofluorescence results also revealed that mJPYZ treatment could reduce the intensity of PKM2 (**Figure 4C**).

In general, PKM2 functions as a metabolic kinase by forming the homotetramer in the cytoplasm. In our study, we discovered that mJPYZ suppressed PKM2 translocating into the nucleus, but increased in the cytoplasm *in vitro*, which might be important for the inhibitory effect of mJPYZ (**Figures 4D, E**). *In vivo*, western blot and immunofluorescence results also supported that mJPYZ decreased the expression of PKM2 (**Figures 4F–H**). Thus, it was confirmed that mJPYZ inhibited aerobic glycolysis in GC cells, which is related to the downregulated nuclear translocation of PKM2 both *in vivo* and *in vitro.*

### Effect of mJPYZ in Gastric Cancer Aerobic Glycolysis Was Largely Dependent on PKM2

**Figure 5:**
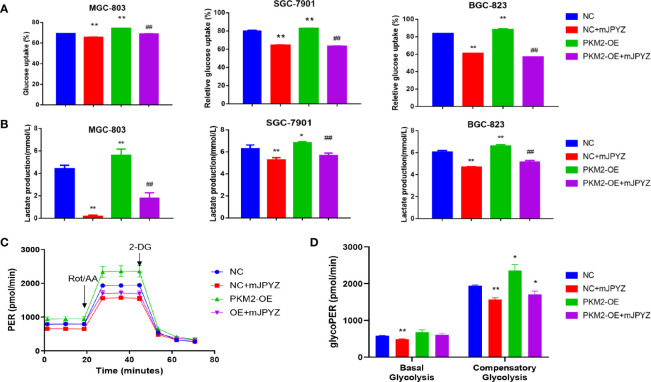
mJPYZ modulated PKM2 and influenced gastric cancer aerobic glycolysis. **(A, B)** The effects of mJPYZ on the glucose uptake and lactate production levels in Negative Control (NC) and PKM2 overexpression gastric cancer cells. **(C, D)** Extracellular acidification rates (ECAR) and Proton Efflux Rate (PER) were measured in gastric cancer cells by Seahorse XFe assays. mJPYZ administration decreased ECARs, PER, glucose consumption and lactate production in NC or PKM2-OE compared with untreated control. Data are represented as mean ± SD, n=3 independent experiments. *P < 0.05, **P < 0.01 versus NC group, ^##^P < 0.01 versus PKM2-OE group by one-way analysis of variance (ANOVA) followed by Tukey’s *post-hoc* tests.

To verify whether mJPYZ reduced glucose uptake and lactate levels were depended on PKM2, we further examined the glucose uptake and lactate levels when PKM2 was over-expression (PKM2-OE) in GC cell lines (MGC-803,SGC-7901,BGC-823). The data showed PKM2-OE group displayed higher glucose consumption and lactate levels than the NC group, while mJPYZ treatment could inhibited GC lactate levels and glucose uptake effectively (**Figures 5A, B**). Consistent with these results, the ECAR and %PER from glycolysis were decreased with mJPYZ treatment, but this phenomenon was reversed by over expression of PKM2 (**Figures 5C, D**). Together, our results suggested that mJPYZ regulated metabolic reprogramming was obviously dependent on PKM2.

### mJPYZ Disrupted the PKM2/HIF-1α Signaling Pathway in GC Cells

**Figure 6:**
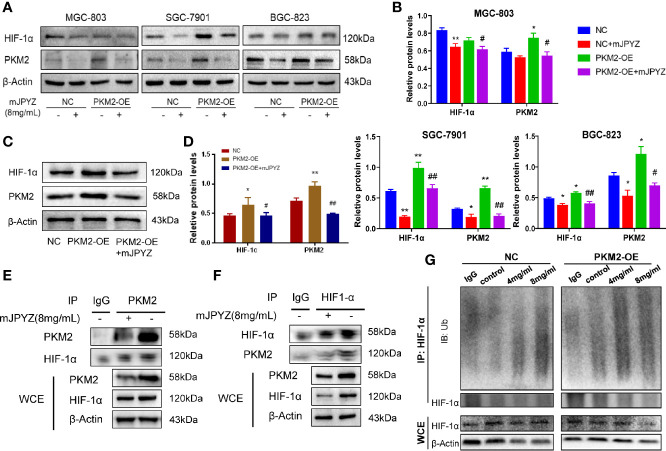
mJPYZ impaired PKM2/HIF-1α signaling pathway. **(A–C)** The effects of PKM2 overexpression on interacted HIF-1α by western blotting. PKM2-OE induced increased PKM2 and HIF-1α, while mJPYZ treatment decreased the expression of PKM2 and HIF-1α whether in NC or PKM2-OE, compared with untreated control. **(D)** Western blotting showed mJPYZ treatment decreased the expression of PKM2 and HIF-1α *in vivo*. **(E, F)** Co-IP analysis of the interaction between PKM2 and HIF1-α in gastric cancer cells. **(G)** mJPYZ treatment increased HIF-1α ubiquitination degradation in a dose-dependently manner. Data are represented as mean ± SD, n=3 independent experiments. *P < 0.05, **P < 0.01 versus NC group, ^#^P < 0.05, ^##^P < 0.01 versus PKM2-OE group by one-way analysis of variance (ANOVA) followed by Tukey’s *post-hoc* tests.

It has been indicated that PKM2 may directly interact with HIF-1α in nuclear and promote transcriptional activation of HIF-1α target genes (15). Therefore, we firstly checked the influence of mJPYZ to HIF-1α in GC cell lines. As expect, our results showed the expression of HIF-1α was significantly suppressed by mJPYZ, and PKM2 could induce the expression of HIF-1α (**Figures 6A, B**). Similarly, we also found mJPYZ inhibited the HIF-1α expression *in vivo* (**Figures 6C, D**). Furthermore, co-immunoprecipitation was then conducted to determine the relationship between PKM2 and HIF-1α in GC cell lines, the results clearly confirmed the strong interaction of HIF-1α and PKM2 (**Figures 6E, F**). Moreover, mJPYZ treatment increased HIF-1α ubiquitination levels in a dose-dependently manner (**Figure 6G**). Above results suggested PKM2/HIF-1α was the key metabolic regulator of mJPYZ in GC cells.

### Overexpression of PKM2 Could Reverse the Inhibitory Effect of mJPYZ on GC Cells Proliferation, Migration and EMT

**Figure 7:**
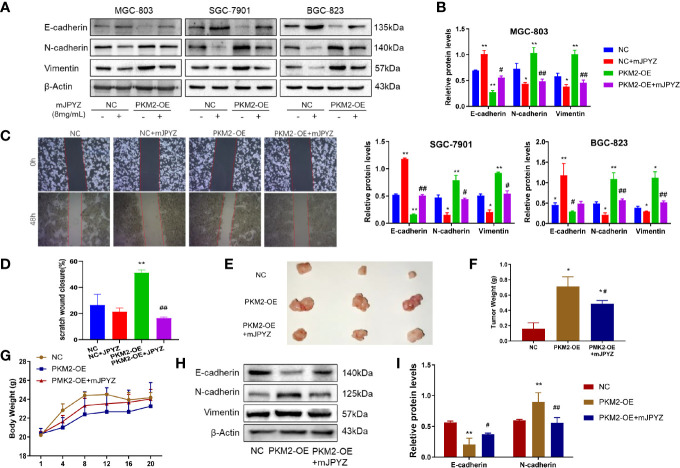
Overexpression of PKM2 reversed the effect of mJPYZ on gastric cancer. **(A, B)** PKM2 affected the effect of mJPYZ on gastric cancer EMT related proteins. **(C, D)** Wound healing assay showed overexpression of PKM2 reversed the mJPYZ influence on migration. **(E–G)** mJPYZ still significantly inhibited the xenograft tumors growth although overexpression of PKM2, while the mice body weight still. **(H, I)** Western blotting analysis of overexpression of PKM2 affected the EMT essential proteins *in vivo*. Data are represented as mean ± SD, n=3 mice or n=3 independent experiments. #P < 0.05, ##P < 0.01 versus PKM2-OE group. *P < 0.05, **P < 0.01 versus control group by one-way analysis of variance (ANOVA) followed by Tukey’s *post-hoc* tests.

Further, we determined the role of PKM2 in migration and EMT of GC cell lines. As shown in **Figure 7A**, the protein levels of N-cadherin and vimentin were enhanced, while the levels of E-cadherin were decreased in the PKM2-OE group, and mJPYZ treatment could reverse it (**Figures 7A, B**). Similarly, we also showed PKM2 overexpression partially reversed the migration inhibitory effect of mJPYZ in GC cells (**Figures 7C, D**). In addition, *in vivo* study revealed that mJPYZ treatment could disrupt PKM2 induced tumor growth. Western blot analysis tissue of GC also indicated that mJPYZ interfered PKM2 overexpression caused EMT related gene expression (**Figures 7E**–**I**). These results further indicated that PKM2 was actually the target of mJPYZ, and mJPYZ induced GC cell growth inhibition and migration were partially regulated by PKM2-mediated glycolysis.

### Chemical Components in mJPYZ and Binding Site Analysis of the mJPYZ-PKM2

**Figure 8:**
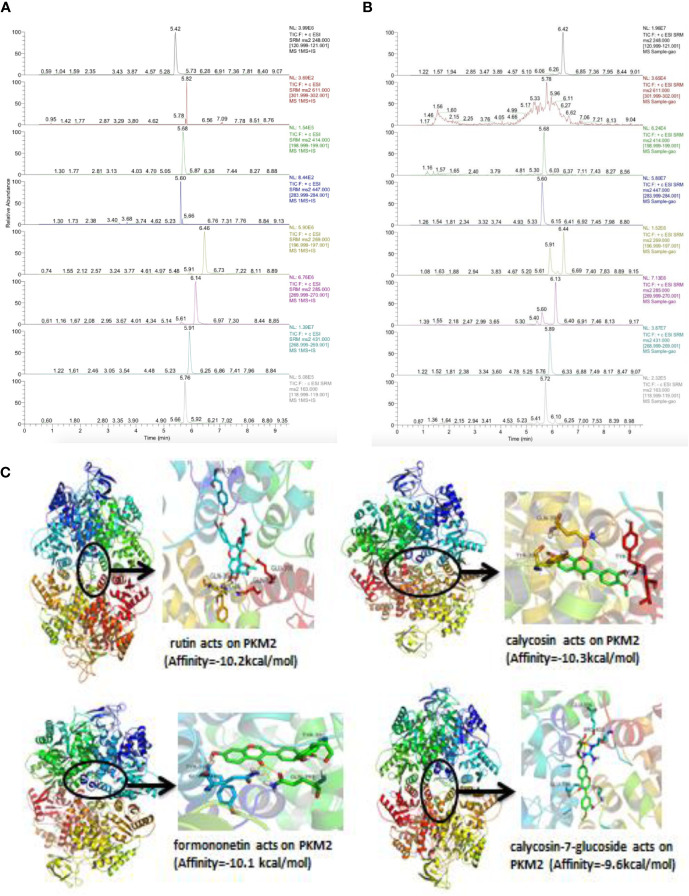
Chemical components in mJPYZ showed strong affinity with PKM2. **(A, B)** The major chemical constituents in mJPYZ and mixture standards were analyzed by LC-ESI-MS/MS analysis. **(C)** Molecular docking of the binding modes of PKM2 with the major compounds in mJPYZ. The results showed rutin, calycosin, formononetin and calycosin-7-glucoside had strong affinity with PKM2.

To investigate whether chemical constituents are involved in the regulation of activation of PKM2 by direct interaction, we performed LC-ESI-MS/MS. The mass spectrometer parameters were: spray voltage, 3 000 eV (-), 4 000 eV (+); capillary temperature, 350℃; shealth gas pressure, 30 psi; auxiliary gas, 10 psi; The ions were detected (selective reaction monitoring) under positive and negative mode with the optimized parameters (**Table S1**). Typical XIC chromatograms of 7 major constituents in mJPYZ and mixture standards are shown in **Figures 8A, B** The contents of rutin, lobetyolin, calycosin-7-glucoside, formononetin, calycosin, ononin and p-coumaric acid in the decoction were finally determined as 0.89 μg/g, 2.01 μg/g, 105.22 μg/g, 25.88 μg/g, 71.26 μg/g, 80.48 μg/g and 19.56 μg/g, respectively. Then we conducted a molecular docking of the binding modes of PKM2 with the 7 compounds. The results showed rutin, calycosin, formononetin and calycosin-7-glucoside had strong affinity with PKM2 (**Figures 8B, C**). These data suggested that chemical compounds from mJPYZ had unerlying binding sites with PKM2 that may influence the activity or stability of PKM2.

## Discussion

Cancer cells undergo complex metabolic regulations and alterations, and targeting metabolic reprogramming refers to a novel therapeutic approaches for cancer treatment (16). Recent studies show chemically synthesized small molecule inhibitors of critical metabolic enzymes are regarded as potentially promising antitumor agents. However, their coexisting non-specific cytotoxicity impairs the proliferation of normal cells, such as immune cells and stem cells, and may lead to severe side effects or drug resistance (17). Therefore, finding or developing more effective and safer drugs or decoctions from herbs is of constructive significance to the development of anticancer drugs. Here, we reported a multi-herb-combined decoction with no obvious cytoxicity, and found mJPYZ significantly reduced the aerobic glycolysis level in GC *in vivo* and *in vitro*, and identified PKM2 is the main target.

As well known, TCM has been widely used a complementary approach for cancer treatment in China over hundreds of years (18). GC is the most prevalent cancer in East Asia (1). Extensive evidence indicated that TCM possesses definite advantages in GC, especially for advanced patients (19, 20). According to the clinical practice, we use the “Qi-invigorating, spleen-strengthening and stasis-removing” method in treating gastric cancer, and obtain a good efficacy in patients (4). mJPYZ as a representative decoction shows obvious advantage for gastric cancer by modulating TAM differentiation (5). In tumor microenvironment, lactate produced by cancer cells has a critical function in TAM polarization (21). Therefore, combined network pharmacology, we speculated that mJPYZ may affect tumor cells glucose metabolic reprogramming by regulating glycolysis. Consequently, we found mJPYZ significantly inhibited gastric cancer cells proliferation and invasion, and reduced the aerobic glycolysis level by dependent on PKM2 in GC cells.

Numerous studies suggest a key role for PKM2 in aerobic glycolysis of cancer. PKM2 is the rate-limiting enzyme that catalyzes the conversion of phosphoenolpyruvate to pyruvate, which is the final step in the glycolytic pathway (22). It has been indicated that PKM2 might be promising molecular target for the treatment of gastric cancer (8). PKM2 exists primarily as an inactive monomer or dimer, when it translocate to the nucleus, which will interact with HIF-1α and regulate expression of numerous proglycolytic enzymes (23). Our study showed mJPYZ inhibited the expression of PKM2, and suppressed PKM2 translocating into the nucleus in GC cells. Moreover, our study also revealed that the overexpression of PKM2 displayed higher glucose consumption and lactate levels. Hence, the inhibition of abnormal PKM2 was critical for the suppression effect of mJPYZ on GC.

As previously mentioned, PKM2 may translocates into the nucleus and forms a complex with HIF-1α to cause an glucose metabolism reprogramming from OXPHOS to glycolysis, as well as facilitates angiogenesis and EMT in cancer by stimulating the target genes (24–26). HIF-1α regulates a variety of tumor processes for adaptation, including metabolism, angiogenesis, invasion and cell proliferation. Studies showed that HIF-1α could modulate various EMT transcription factors, histone modifiers, enzymes (MMPs), chemokine receptors (27). Therefore, we checked the effect of mJPYZ to HIF-1α in GC cells, and observed that decreased expression level of HIF-1α when mJPYZ treatment. The positive correlation between PKM2 and HIF-1α was also confirmed by PKM2-OE. In agreement with previous reports, we verified that HIF-1α could interact with PKM2 using Co-IP in GC cells. PKM2 lead to a positive feedback loop that amplifies HIF-1 activity and may enhance metabolic reprogramming. Prolyl-hydroxylated HIF-1α binds to the von Hippel-Lindau (VHL) tumor suppressor protein, which recruits the E3- ubiquitin-ligase complex and causes proteasome degradation of HIF-1α (26). Thus, HIF-1α ubiquitination degradation is the key to blocking downstream signal activation. Here we identified that mJPYZ treatment increased HIF-1α ubiquitination levels in a dose-dependently manner.

## Conclusion

In summary, our present study suggested that high expression of PKM2 is required for maintaining the malignant phenotype of GC cells. The TCM decoction mJPYZ inhibited GC cells growth and EMT by reducing of glycolysis in PKM2/HIF-1α signaling-dependent manner. This evidence expanded our understanding of the anti-tumor mechanism of mJPYZ and further indicated mJPYZ a potential anti-tumor agent for GC patients.

## Data Availability Statement

The original contributions presented in the study are included in the article/**Supplementary Material**. Further inquiries can be directed to the corresponding author.

## Ethics Statement

The animal study was reviewed and approved by The Experimental Animal Ethics Committee of Nanjing Medical University.

## Author Contributions

QS and JW conceptualized the research hypothesis and designed the study. MY, HXW, XZ, RZ, HDW, XC, and MZ performed the laboratory experiments. MY and HXW performed the statistical analysis and data interpretation. QS, JW, and MY interpreted the results and wrote the manuscript. All authors performed critical revision of the final manuscript. SL supervised the study. All authors contributed to the article and approved the submitted version.

## Funding

This study was supported by National Natural Science Foundation of China (No. 81973609, 81973782, 82174197, 81704031), Science and Technology Planning Project of Jiangsu Province, China (No. BK20211392), Jiangsu Provincial Medical Youth Talent (QNRC2016641), “333” Project of Jiangsu Province (LGY2018065), Jiangsu Provincial Hospital of Traditional Chinese Medicine Academic Talent Program (Y2018RC33); A Project Funded by the Priority Academic Program Development of Jiangsu Higher Education Institutions (PAPD), The Open Projects of the Discipline of Chinese Medicine of Nanjing University of Chinese Medicine Supported by the Subject of Academic priority discipline of Jiangsu Higher Education Institutions (ZYX03KF019, ZYX03KF021, ZYX03KF029). Postgraduate Research &Practice Innovation Program of Jiangsu Province (SJCX21_0692).

## Conflict of Interest

The authors declare that the research was conducted in the absence of any commercial or financial relationships that could be construed as a potential conflict of interest.

## Publisher’s Note

All claims expressed in this article are solely those of the authors and do not necessarily represent those of their affiliated organizations, or those of the publisher, the editors and the reviewers. Any product that may be evaluated in this article, or claim that may be made by its manufacturer, is not guaranteed or endorsed by the publisher.
