# Supplementary material for: PKM2 Is the Target of a Multi-Herb-Combined Decoction During the Inhibition of Gastric Cancer Progression
Source: Front Oncol. 2021 Dec 2;11:767116. doi: 10.3389/fonc.2021.767116 (PMC8675178; doi:10.3389/fonc.2021.767116)
Supplement: Supplementary file 1 [file DataSheet_1.docx]

**Supplementary Table 1 LC-MS/MS parameters optimized for the target analytes**

| **Chemical constituents** | **Parent (m/z)** | **Product**  **(m/z)** | **Collision energy**  **(eV)** | **Tube lens** |
| --- | --- | --- | --- | --- |
| Rutin | 611.11 | 302.96 | 22 | 216 |
| Lobetyolin | 414.14 | 199.09 | 8 | 228 |
| Calycosin-7-glucoside | 447.06 | 284.99 | 17 | 175 |
| Formononetin | 269.01 | 197.03 | 39 | 205 |
| Calycosin | 285.01 | 270.00 | 22 | 123 |
| Ononin | 431.06 | 269.01 | 19 | 168 |
| P-coumaric acid | 162.98 | 119.13 | 20 | 105 |

**Supplementary Figure 1 Viability of gastric epithelial cell (GES-1) interfered by mJPYZ.** Gastric epithelial cell proliferation interfered with mJPYZ in a dose- and time-dependent manner and found there is no affection.
